# Catching the Main Ethiopian Rift evolving towards plate divergence

**DOI:** 10.1038/s41598-021-01259-6

**Published:** 2021-11-08

**Authors:** Eugenio Nicotra, Marco Viccaro, Paola Donato, Valerio Acocella, Rosanna De Rosa

**Affiliations:** 1 Dipartimento di Biologia Ecologia e Scienze della Terra, Università della Calabria, Arcavacata di Rende, Cosenza, Italy; 2 Dipartimento di Scienze Biologiche Geologiche e Ambientali, Università di Catania, Catania, Italy; 3 Sezione di Catania-Osservatorio Etneo, Istituto Nazionale di Geofisica e Vulcanologia, Catania, Italy; 4 Dipartimento di Scienze, Università di Roma Tre, Rome, Italy

**Keywords:** Petrology, Tectonics, Volcanology

## Abstract

Magmatism accompanies rifting along divergent plate boundaries, although its role before continental breakup remains poorly understood. For example, the magma-assisted Northern Main Ethiopian Rift (NMER) lacks current volcanism and clear tectono-magmatic relationships with its contiguous rift portions. Here we define its magmatic behaviour, identifying the most recent eruptive fissures (EF) whose aphyric basalts have a higher Ti content than those of older monogenetic scoria cones (MSC), which are porphyritic and plagioclase-dominated. Despite these differences, calculations highlight a similar parental melt for EF and MSC products, suggesting only a different evolutionary history after melt generation. While MSC magmas underwent a further step of storage at intermediate crustal levels, EF magmas rose directly from the base of the crust without contamination, even below older polygenetic volcanoes, suggesting rapid propagation of transcrustal dikes across solidified magma chambers. Whether this recent condition in the NMER is stable or transient, it indicates a transition from central polygenetic to linear fissure volcanism, indicative of increased tensile conditions and volcanism directly fed from the base of the crust, suggesting transition towards mature rifting.

## Introduction

**Figure 1:**
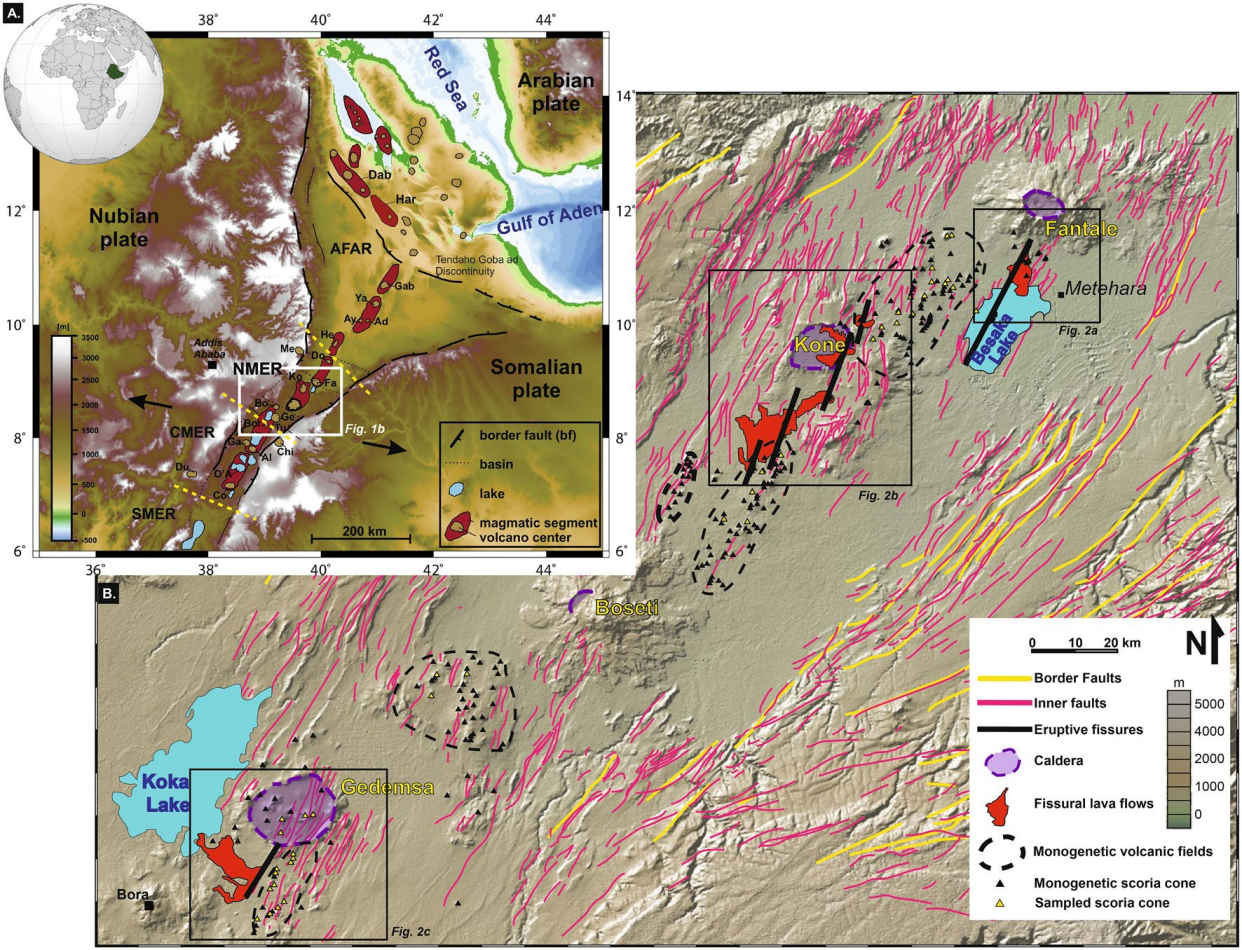
Digital terrain model (DTM) and digital elevation model (DEM) of the Afar and Northern Main Ethiopian Rift.** (A)** DTM of Afar and Main Ethiopian Rift (after^64^). Reddish ellipses represent the different tectono-magmatic systems, arranged én-échélon. The NMER lies within the red square. *F-D* Fantale-Doffen segment, *K-B* Kone-Boseti segment, *Ko* Koka segment, *TGD* Tendaho-Goba’ad discontinuity, *Dah* Dabbahu, *Har* Manda Hararo, *Ay* Ayelu-Amoissa, *Co* Corbetti. (**B)** DEM of the Northern Main Ethiopian Rift, highlighting the main volcano-tectonic structures (map taken from the freeware GeoMapApp software v. 3.6.14; http://www.geomapapp.org/). Faults are taken from^65^, whereas monogenetic scoria cones (black triangles) have been manually picked up on the DEM and double-checked on Google Earth software (http://earth.google.com). Yellow triangles represent the sampled MSC, whereas black empty squares are referred to the area depicted in Fig. 2.

The immature stages of rifting before continental breakup can be best investigated at the Main Ethiopian Rift (MER)-Afar system (Fig. 1A), characterized by the northward increase in the extension and magmatic activity, from incipient continental rifting in the Southern MER, to continental breakup in Afar^1,2^. The Northern MER (NMER) extends between Gedemsa and Dofen central volcanoes and the contiguous Central-Southern MER and Afar at north (Fig. 1A) and shows apparently controversial features. Here, a melt-induced anisotropy in the upper mantle supports magma-assisted rifting^3–5^, culminating in polygenetic volcanoes with calderas and ignimbrites, and hundreds of monogenetic volcanoes. These features are at odds with the negligible recent volcanic activity of the NMER, where the last eruption occurred in the nineteenth century, and the central volcanoes in the last decades have not shown evident unrest^6–9^. It is unclear how these features reconcile with the magmatic activity occurring in the contiguous portions of the MER (at south) and Afar (at north), which show evidence of central and linear magmatic activity, respectively. In the Central and Southern MER, volcanic activity shows predominant central volcanoes often experiencing unrest, as at Corbetti, Aluto and Bora^7,10,11^ (Fig. 1A). In the southern region of Afar the predominant linear magmatic activity is underlined by dike-induced recent unrest at Hertali, Ayelu-Amoissa and Manda Hararo^2,12–14^ (Fig. 1A). The poorly understood and apparently controversial magmatic features of the NMER hinder properly defining the role of magma on continental rifting and, in turn, the maturity of this portion of the rift, which remain both elusive. Here we contribute to solve this conundrum by reconstructing the recent magmatic activity of the NMER considering its widespread monogenetic volcanism (Fig. 1).

## Tectono-magmatic background

Mio-Pliocene continental rifting activated the border faults of the MER and was accompanied by diffuse magmatic activity. During Quaternary, tectonic and volcanic activity focused on magmatic systems within the rift, partially deactivating the border faults (Fig. 1)^1^. Magmatic systems are focused zones of extension and volcanic activity, parallel to the rift axis, accommodating > 80% of the extensional strain at depth < 10 km, with each system associated with a polygenetic felsic volcano and several mafic monogenetic vents^15–17^. In addition, magmatic systems induce rift opening through dike intrusions feeding the monogenetic vents^16,18–20^. Volcanic activity along the MER is mirrored by a similar segmentation of high-velocity bodies at 10–15 km of depth, interpreted as cooled mafic intrusions^5,21,22^.

Fantale, Kone, Boseti and Gedemsa, each located a few tens of km apart along the rift axis, are the main polygenetic central volcanoes of the NMER (Fig. 1), formed by successions of trachytic to rhyolitic lava flows and ignimbrite deposits and associated with one or more caldera collapse episodes. Not being the main focus of this work, we refer to literature for their characterization (e.g.,^18,23–31^).

**Figure 2:**
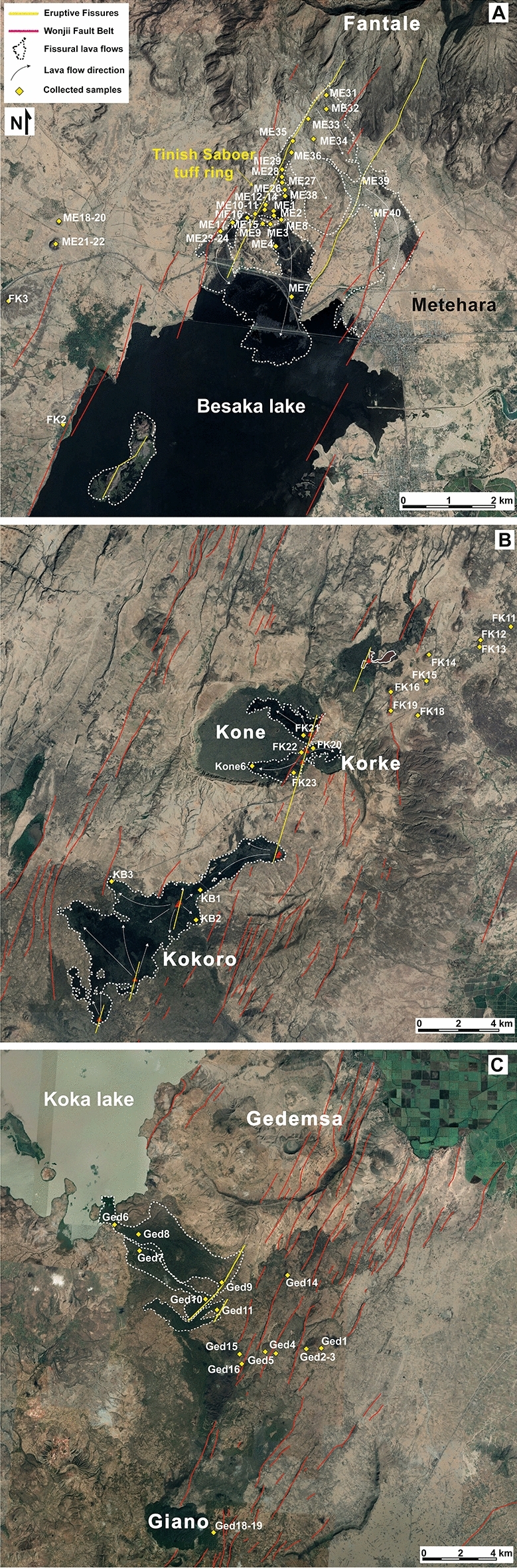
Detail of the areas of the main eruptive fissures found in the Northern Main Ethiopian Rift.** (A)** Satellite view (map taken from Google Earth, date of acquisition 1/11/2019; http://earth.google.com) of the area of the southern flank of Fantale volcano, where two parallel Eruptive Fissures (EF) developed next to Metehara village (yellow lines) feeding two small lava flows toward south, into Lake Besaka; the westernmost fissure continued also into the Lake, feeding a small chain of scoria cones. In the western portion of the area, four Monogenetic Scoria Cones crop out (ME18-22 and FK2-3 samples). (**B)** Satellite images (map taken from Google Earth, date of acquisition 1/11/2019; http://earth.google.com) of the area of Kone volcano. Here, the 1810–20 CE eruption developed several eruptive fissures, from which modest lava flow fields formed within the Kone caldera and in the area of Kokoro. (**C)** Satellite images (map taken from Google Earth, date of acquisition 12/23/2019; http://earth.google.com) of the area south of Gedemsa volcano and of Koka Lake.

Mafic monogenetic volcanoes focus along the strike of the magmatic systems, reaching the highest density in the NMER. Here, at least 283 (cf. “Methods”; Figs. 1, 2) monogenetic volcanoes can be recognized. These mainly consist of basaltic scoria cones, at times associated with minor lava flows and commonly generating short (< 4 km long) alignments parallel to the strike of the magmatic system.

## Results

### Geology

We surveyed and sampled 41 of the 283 recognized monogenetic scoria and spatter cones (MSC) outcropping in the Northern Main Ethiopian Rift (NMER), between Fantale and Gedemsa central volcanoes [Figs. 1 and 2; Electronic Supplementary Material (ESM) 1]. The term “monogenetic” refers here to all the volcanic edifices (e.g., small-volume spatter and scoria cones, maars and small lava domes) produced by a single eruption. The MSC cluster into 5 major volcanic fields (dotted ellipses in Fig. 1B) along approximately NNE-SSW elongated areas between the major central volcanoes (Fig. 1B). Approximately 216 out of the 283 cones can be grouped into 39 alignments (cf. Methods section), whereas 67 cones remain apparently isolated (Fig. 1). In particular: a) 222 MSC have been recognized between Fantale and Boseti (ca. 5 cones per 10 km^2^^32^), of which 178 grouped into 30 eruptive episodes (cf. Methods); b) 61 MSC have been recognized between Boseti and Giano (Fig. 1), of which 38 grouped into 9 eruptive episodes. These alignments are parallel to the rift direction and their length ranges between 0.3 and 3.6 km (average of ca. 1.67 km). Although the age of the MSC is poorly constrained, most of them are generally coeval to the Late Quaternary activity of the central volcanoes, with the northernmost cones being covered by the ~ 168 ka Fantale ignimbrite^27^ and other monogenetic cones to the south being sutured by Holocene epiclastic deposits.

In the NMER, we have also distinguished three Eruptive Fissures (EF; Fig. 2), which differ from the volcano-tectonic lineaments giving monogenetic scoria cones: (a) the eruptive fissures form the most extended alignments of cones, 6 to 14 km long, belonging to the same eruptive event, recognized through remote sensing data and in the field; (b) the eruptive fissures are neither eroded nor partially buried and, in the field, their products are generally massive and mostly aphyric, without evidence of alteration or weathering; (c) the eruptive fissures are accompanied by a relatively larger (1–2 orders of magnitude more voluminous) amount of lava with respect to the monogenetic scoria cones.

The northernmost eruptive fissure (herein Metehara EF) is formed by two parallel and én-échelon segments (11 and 7 km long), extending from the middle (ca. up to 500 m above the rift floor) southern slope of Fantale volcano to the scoria cones inside Lake Besaka (Fig. 2A). At its northernmost tip, the eruptive fissure does not affect the rim of Fantale caldera. Along the Metehara EF a chain of scoria cones and a small-volume (~ 53 × 10^6^ m^3^^8^) lava field formed (Fig. 2A). These products have been previously associated with the two post-caldera lava flows within the caldera (e.g.^33^). Nonetheless, our survey reveals that intra-caldera lava flows are obsidian coulee, trachytic-rhyolitic in composition and without macroscopic evidence of magma mingling/mixing (Fa1 and Fa2 samples in ESM 1 and 2; cf. also^27^), as also recently confirmed by^31^. Historical reports and local oral traditions date this eruptive event at 1810 CE^8,34,35^.

The eruptive fissure affecting the Kone volcanic complex (Kone EF) consists of 5 main én-échelon segments, for a total length of 25 km (Figs. 1, 2). The main fissure (EFKone-1 in Fig. 2B) is ~7 km long and passes through the rims of the two main Kone calderas (Fig. 2B). Our survey has highlighted that the most recent lava flows filling the Kone-Korke calderas were fed by three scoria cones developed along the EFKone-1 fissure system (Fig. 2B), which continues to the south forming two small scoria cones on the southern lower flank of Kone. The southernmost termination of the Kone eruptive fissure lies south of the calderas, in the Kokoro area (Fig. 2B), where an én-echelon segment with offset of ~3 km produced a modest lava flow (Kb1-3 samples in Fig. 2 and ESM 1 and 2), consistent with previous studies^27,29^. The Kone EF is dated at 1810-20 CE^8,23,29,36^.

The third identified eruptive fissure (Koka EF) lies just outside the southern rim of Gedemsa caldera (Fig. 2C). It extends for ~ 7 km and it is arranged in two én-échélon N10°E segments, feeding a mostly aphyric and non-altered lava that flowed into Koka Lake (Fig. 2C). This eruptive event, coinciding with the Melkasa Unit of^37^, appears distinct from that generating the products emitted within the Gedemsa caldera. Although an absolute age is not available, according to historical reports this eruptive fissure may have formed during 1775 ± 25 CE, when this area was volcanically active^8^.

### Petrography and mineral chemistry

Products of monogenetic scoria cones basalts all display seriate porphyritic textures, with Porphyritic Index (P.I.) between 15 and 35 vol.%.

**Figure 3:**
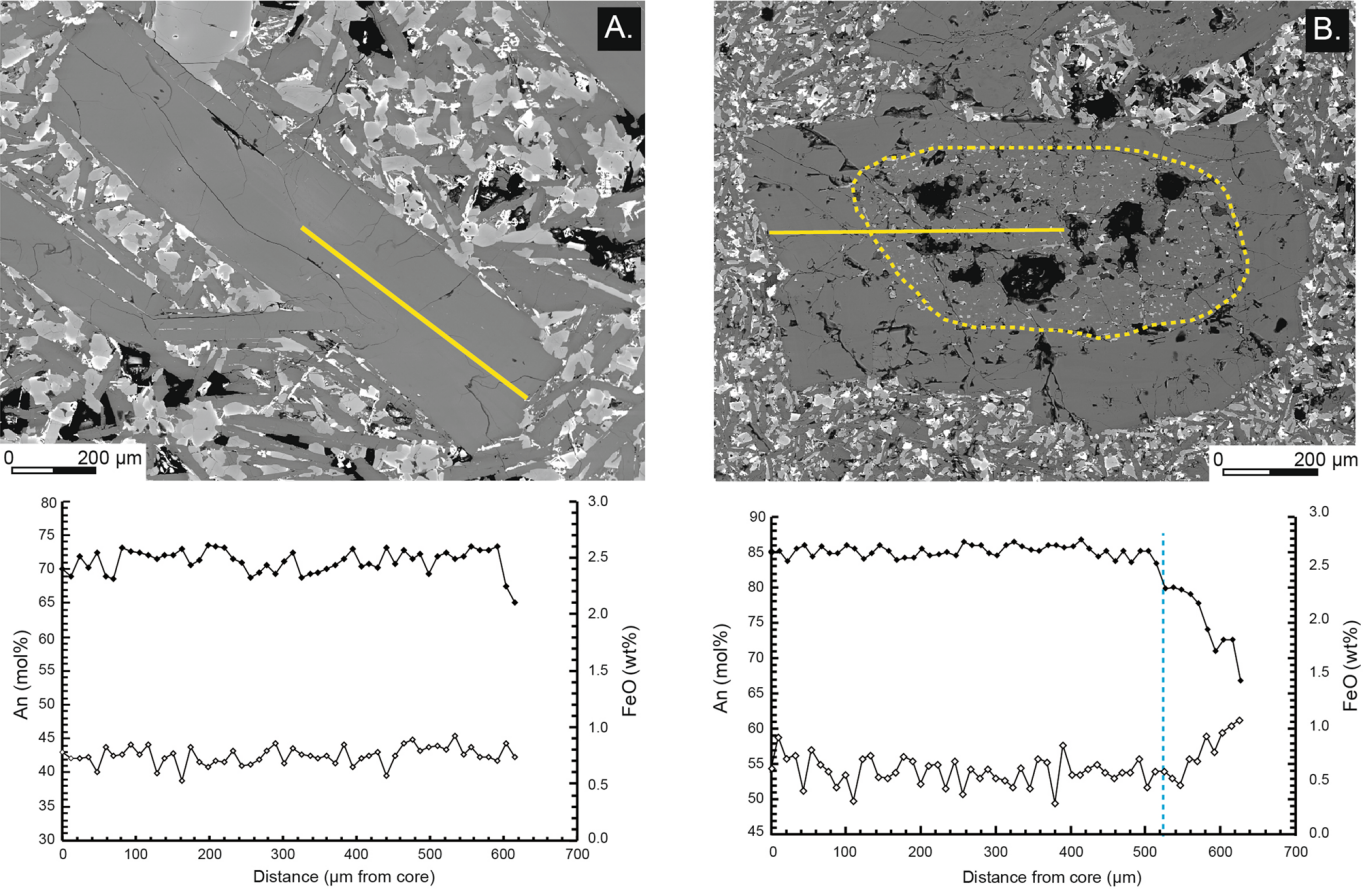
Back-scattered electron images acquired through SEM and compositional core-to-rim profile of selected plagioclase crystals of MSC (no phenocrysts exist in EF).** (A)** Plagioclase with oscillatory zoning pattern and small amplitude variations of An contents. (**B)** Plagioclase with dissolved core texture, with a typical rounded core marked by a ΔAn_5–9_ decrease at fairly constant FeO contents.

Phenocrysts of An_76-89_ plagioclase are dominant (40–90% of the total crystal volume), with size up to 4 mm. Textural and microanalytical observations on MSC plagioclase highlight prevalent (91%) oscillatory-zoned crystals, with oscillations varying from ΔAn_1–3_ to ΔAn_3–7_ (Fig. 3A; ESM 3). The plagioclase suite is completed (~ 9%) by phenocrysts with dissolved cores, i.e. zones of resorption often causing core rounding. Compositional profiles denote marked An decrease (> ΔAn_15_) at fairly constant FeO content at the boundary of the rounded core (Fig. 3B; ESM 3). Phenocrysts of Fo_78-89_ olivine (10–35 vol.%), augitic clinopyroxene (0–10 vol.%), orthopyroxene and opaque oxide (together < 5 vol.%) complete the mineral assemblage. The same phases also occur in the groundmass, which is from vitrophyric to intersertal. All analysed samples exhibit glomerophyric structures generally formed by plagioclase (80–90 vol.%), olivine (5–10 vol.%) and augitic clinopyroxene (< 5 vol.%), with similar composition to the phenocrysts.

Rock samples of the three analysed eruptive fissures (i.e., EF) are mostly aphyric (P.I.: Metehara and Kone-Kokoro 0–2 vol.%; Koka = 3–5 vol.%). Their rare phenocrysts are mainly euhedral An_72-89_ (at core) to An_67-80_ (rims; ESM 3) plagioclase crystals, with either prismatic or acicular habitus, and Fe-Ti oxides. Compositional oscillatory zoning is the most frequent texture of the plagioclases (98% of the total crystal amount), with ΔAn ranging between ΔAn_1–3_ to ΔAn_3–7_ (ESM 3). Plagioclases presenting resorption are very rare (< 2%) in EF basalts. Some EF samples also display a few altered xenocrysts of olivine, clinopyroxene and orthopyroxene. Groundmass is generally very homogeneous, being intersertal, with microlites of plagioclase, olivine and orthopyroxene. No enclaves or evidence for magma mixing have been detected within the EF basalts of Metehara, Kone-Kokoro and Koka.

### Whole rock geochemistry

**Figure 4:**
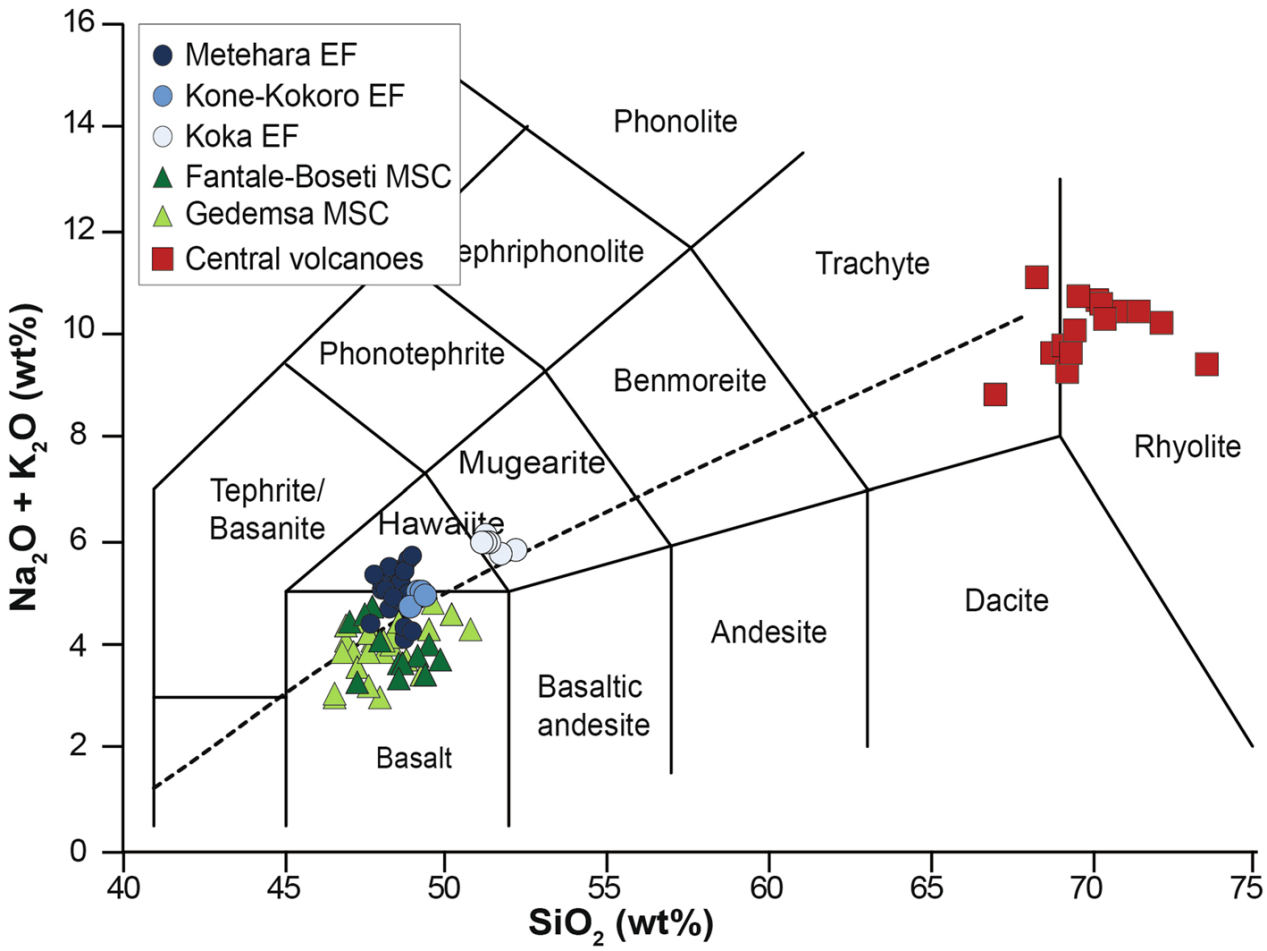
Total alkali silica (TAS) diagram of analysed products. Total alkali vs. silica (TAS) diagram for monogenetic scoria cones (MSC), eruptive fissures (EF) and central volcanoes products. Whole-rock compositions for MSC of the Gedemsa area have been also included^40,41^.

**Figure 5:**
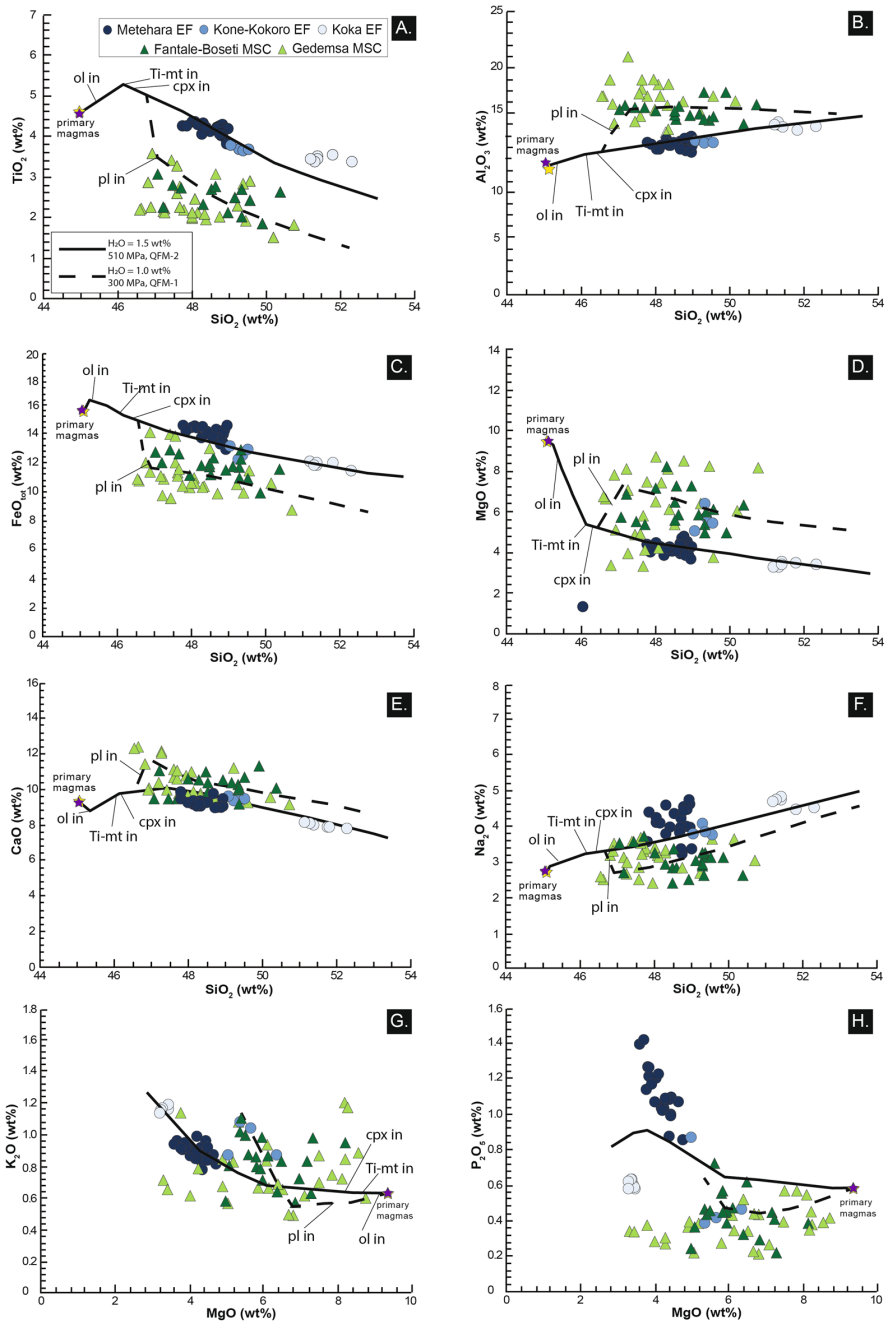
Whole rock compositions for major elements of analysed products. Samples labelled “Gedemsa MSC” are taken from^41^. SiO_2_ and MgO vs. major elements variation diagrams for the analysed products (compositions in ESM 2). EF products show a peculiar high-TiO_2_–FeO_tot_–Na_2_O–P_2_O_5_ signature **(A,C,F,H)**. The yellow and black stars represent the parental (and primary) magma composition for both EF and MSC products calculated by means of the PRIMELT3 software^62^. Liquid lines of descent obtained from Rhyolite-MELTS crystal fractionation simulations^47^ are also shown; solid and dotted lines represent two different conditions of crystallization, respectively at: (I) temperature between the eutectic temperature of 1331 °C (obtained by Rhyolite-MELTS, in accordance with the interval 1300–1350 °C found by^65,66^) and 1000 °C; H_2_O = 1.5 wt.%, 510 MPa, QFM-2 and (II) T = 1151–1000 °C; H_2_O = 1 wt.%, 300 MPa, QFM-1. MSC products, although with less homogeneous compositions, underwent two processes of crystal fractionation at different pressures before to be erupted.

**Figure 6:**
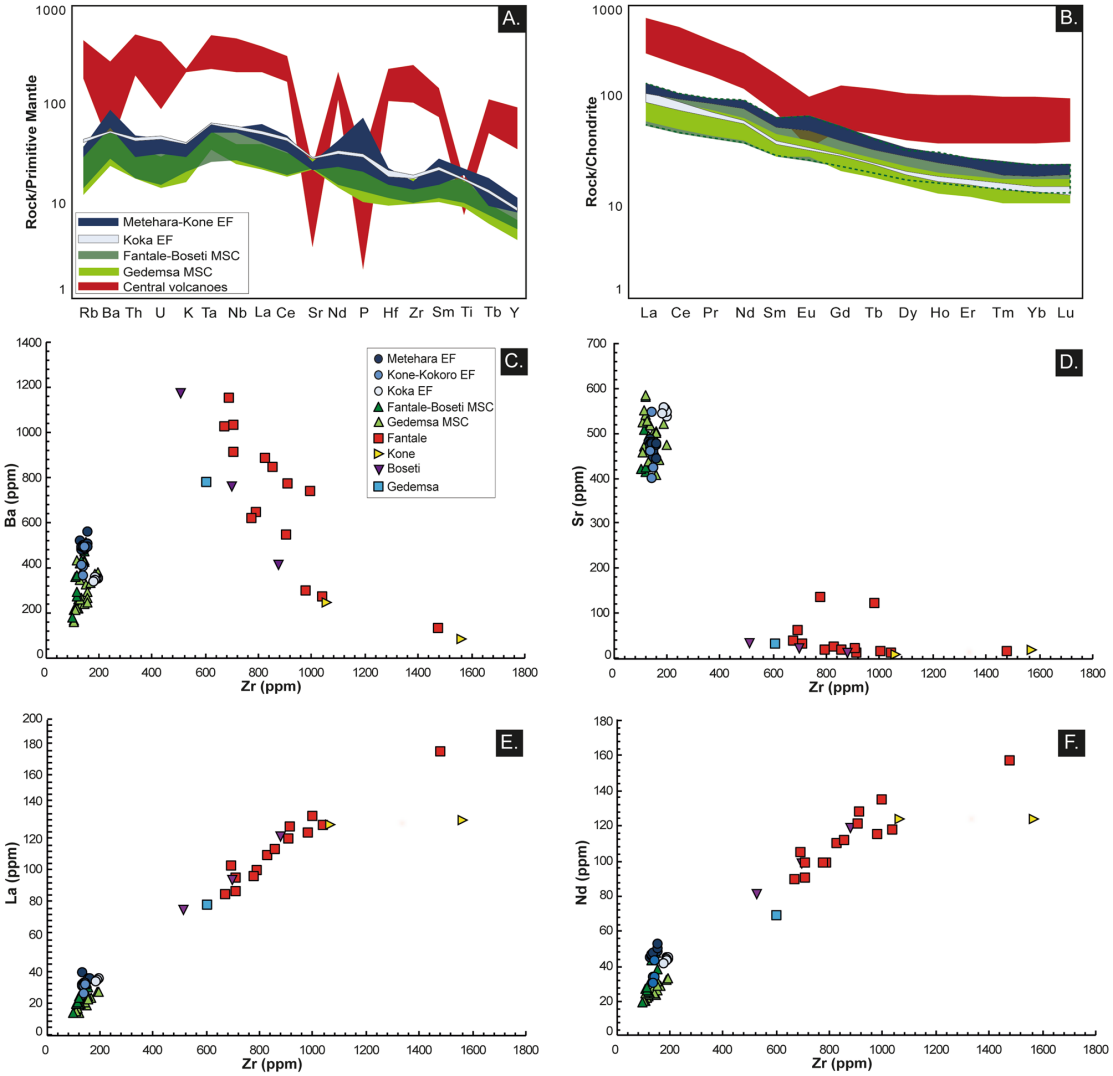
Normalized spider and selected trace element diagrams. (**A,B)** Spider and REE diagrams for the analysed products. Compositions of central volcanoes could be also one order of magnitude more enriched with respect to the EF and MSC products. Notably, central volcanoes present deep negative anomalies in Sr and P and a slight negative anomaly in Eu, suggesting prominent fractionation processes involving plagioclase and apatite. (**C–F)** Zr vs. selected trace element diagrams for the entire dataset, including central volcanoes. No mixing trends can be observed between basic (EF + MSC) and rhyolitic products.

**Figure 7:**
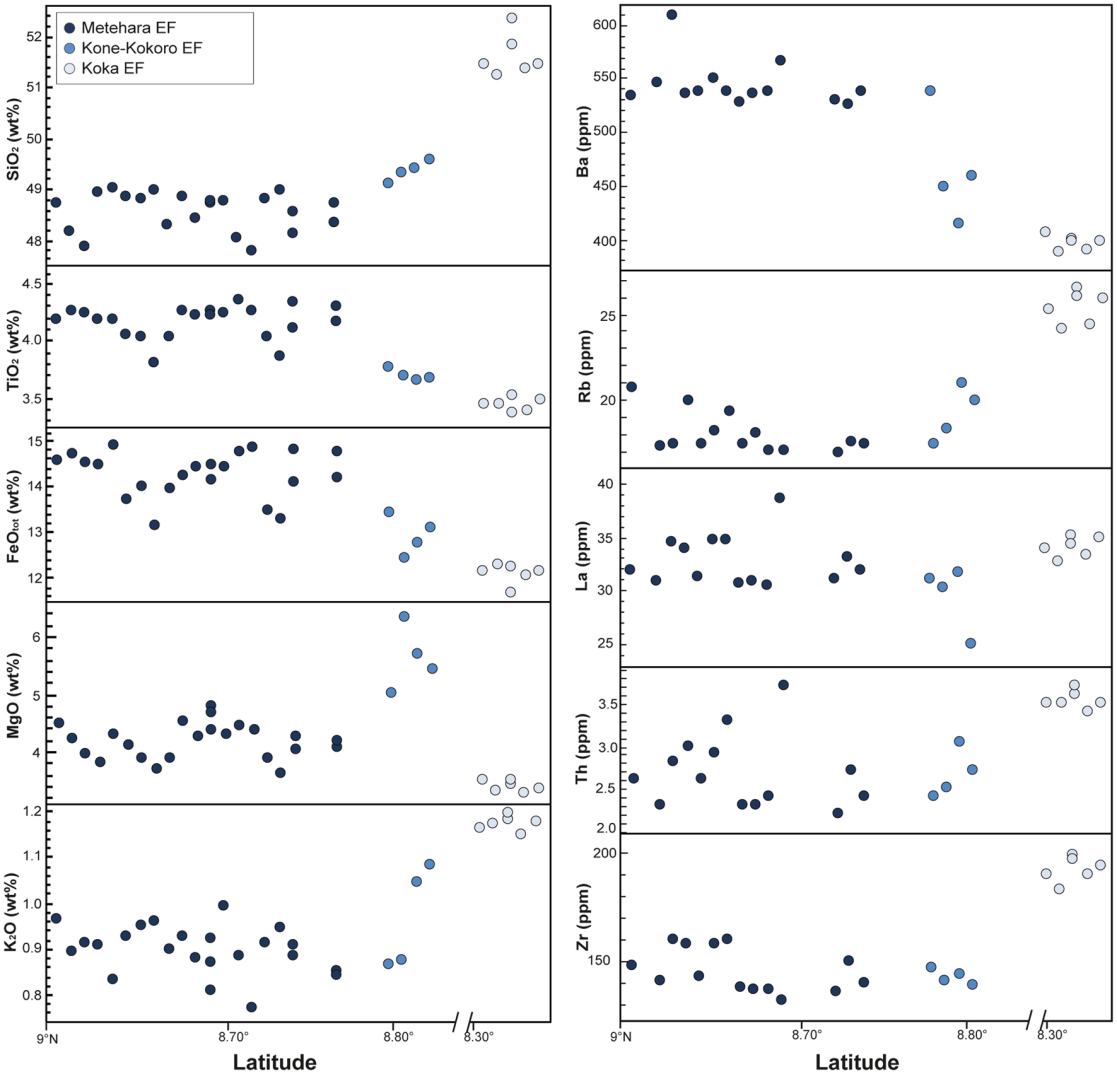
Latitude vs. major and trace element whole rock compositions for the eruptive fissures (EF) of Metehara, Kone-Kokoro and Koka.

Whole rock compositions show that magmas of the Monogenetic Scoria Cones (MSC) are distinct from those of the more recent Eruptive Fissures (EF) of Metehara, Kone-Kokoro and Koka Lake (Figs. 4, 5, 6, and 7; ESM 2).

MSC plot in the field of basalts (Fig. 4A; ESM 2), exhibiting wide ranges for most of major elements (e.g., TiO_2_ 1.5–3.4 wt.%; FeO 8.8–14.1 wt.%; MgO 3.2–8.2 wt.%; Fig. 5B,D,E; ESM 2). This variability is reflected by most of their trace element contents (e.g., Fig. 6; ESM 2) and their normalized patterns for rare earth and other incompatible trace elements (except for Sr; Fig. 6A,B; ESM 2). With respect to the rock classification for Ethiopian high-Ti volcanic products proposed by^38^, MSC basalts have an almost full fit with the whole rock range compositions of HT1 basalts, except for slightly lower Y values. No variability in whole rock compositions has been observed as a function of the MSC latitude (cf. ESM 2).

The EF products of Metehara and Kone-Kokoro present a more homogeneous composition, being basalts and hawaiites (Fig. 4), whereas Koka samples plot at the boundary between hawaiites and mugearites (Fig. 5; ESM 2). All eruptive fissures products are distinctively enriched in TiO_2_ (up to 4.3 wt.%), FeO_tot_, Na_2_O, P_2_O_5_ and depleted in Al_2_O_3_ with respect to MSC (Fig. 5B–D,G–H; ESM 2), with some variations related to their latitude (Fig. 7; ESM 2). Similar relationships with latitude are shown by some trace elements, with the southernmost samples (Koka EF) richer in Rb and Zr and poorer in Ba (Fig. 7; ESM 2). EF rocks present a good fit with the compositional range of HT2 basalts^38^, although with higher values of SiO_2_, P_2_O_5_ and Y.

With the exception of a comenditic lava flowing southeast of Gedemsa, all analysed products of central volcanoes are peralkaline trachytes and rhyolites (i.e., pantellerites; Fig. 4; ESM 2). Comparison of the compositions of our 20 samples from the central volcanoes to those of their three eruptive fissures suggests lack of any mixing (Fig. 6C–F), with strong fractionation of plagioclase and in less extent of apatite and Ti-oxides, respectively highlighted by the deep negative anomalies of Ba, Sr, P and Ti in the incompatible trace element diagrams and the negative Eu anomaly (Fig. 6A,B).

### Modelling the plumbing system of the NMER

Integrating geological, geochemical and petrographic data indicates that volcanism of Monogenetic Scoria Cones (MSC) presents systematic differences from those resulting in the Eruptive Fissures (EF): (a) the fissures associated with MSC have maximum length of ~ 4 km, whereas the fissures of the EF may reach up to ~ 14 km; (b) the EF mark the most recent eruptive episode in the NMER; (c) the MSC erupt a lower volume of products (1–2 orders less) than the EF; (d) the MSC magmas typically present a whole rock signature with lower contents of TiO_2_, FeO_tot_, Na_2_O and P_2_O_5_ and higher of Al_2_O_3_ than the EF magmas; e) the MSC products are porphyritic, with the mineralogical assemblage being plagioclase-dominated, whereas the EF products are almost aphyric (P.I. = 0–5 vol.%). These different features could be either attributed to small changes in the partial melting processes occurring at depth within the rift, producing two different parental magmas, or to distinct conditions experienced by the same parental magma during ascent.

In order to discern between these two possibilities, the primary magma compositions for products of MSC and EF have been obtained through a mass balance calculation approach able to reset the modifications of whole rock composition due to crystal fractionation (cf. Methods). Resulting compositions show that MSC and EF basalts derive from parental magmas with similar petrological and geochemical features (overlapped star symbols in Fig. 5). This supports previous studies on primary magma at the MER^39,40^. To obtain primitive compositions for the MSC lavas, addition of at least 27 vol.% of a solid fraction (Pl 10%, Cpx 9%, Ol 6%, Ti-mt 2%) is required. Conversely, magmas feeding the recent EF are in equilibrium with the mantle adding a total solid fraction of at least 20 vol.% of a mafic assemblage (Ol 9%, Cpx 8%, Ti-Mt 3%). The main difference between the two crystallization processes is the presence of plagioclase (partially fractionated, and mostly erupted) only in the MSC magmas, whereas the other mineralogical phases are generally comparable.

Differences between MSC and EF should therefore be related to varying modes of magma storage and ascent. Petrography of MSC and EF products suggests different chemical and physical conditions and processes of magma storage and ascent. The plagioclase dominance in the mildly porphyritic (P.I. 15–35 vol.%) Monogenetic Scoria Cones products suggest storage at the favourable P–T conditions for this mineralogical phase, i.e. in the shallow crust. Conversely, the absence of crystals (P.I. 0–5 vol.%) and the lack of negative Eu anomalies in the spider-diagrams of Metehara, Kone-Kokoro and Koka EF (Fig. 6A,B) suggest that these magmas experienced direct ascent from a deep reservoir located below the nucleation depth of plagioclase (~ 10 km for NMER; cf. text below).

In the MER, differentiation (mainly crystal fractionation plus assimilation) has been primarily used to explain the silica-rich peralkaline composition of central volcanoes and the “Daly-gap”, which is the spatial coincidence of basaltic and rhyolitic products (e.g.^10,18,20,41–43^). Conversely, here we consider the wider process of crustal magma differentiation (since the onset of magma generation), also including the deeper portion of the plumbing systems (19–25 km in the NMER^44^), where geophysical investigations have detected up to 4% of melt^21,45^.

Simulations of crystal fractionation for EF and MSC magmas are performed through the thermodynamic-based algorithm of Rhyolite-MELTS (cf. “Methods”^46,47^). Considering that only poorly-differentiated compositions are involved in the two sample suites, our Rhyolite-MELTS modelling is based on two different (and simplified) configurations of magma plumbing systems: (i) one step of deep isobaric crystallization, common to all products, and (ii) one step of intermediate-shallow crystallization. All the simulations start from the calculated primary liquid composition for NMER magmas, which is fairly the same for MSC and EF and is representative for a magma whose composition is not modified by differentiation processes (cf. text above and purple/yellow stars in Fig. 5; ESM 2).

Deep and isobaric crystallization is simulated through Rhyolite-MELTS by means of ca. 250 runs, within the ranges of parameters shown in Table 1. Pressures of crystallization are tested between 350 and 900 MPa, considering adiabatic magma ascent from the source of partial melting to a deep storage reservoir. Oxidation state is tested between QFM-2 and QFM + 1, representing a possible range for MER magmas at different depths^39^. For the initial H_2_O content, we consider a spectrum from a fairly undegassed to a poorly degassed basaltic magma (1–2 wt.%; cf.^42^). Results show that the model characterized by the parameters listed in Table 1 presents a best-fit with the compositions of EF products (solid lines in Figs. 5B–H). The obtained pressure of 510 MPa, corresponding to ~ 19 km of depth (assuming an average density of 2800 kg/m^3^^48^) and the H_2_O content of 1.5 wt.% fully match the values provided by the deepest and undegassed olivine-hosted melt inclusions in the NMER^40^. The Quartz-Fayalite-Magnetite (QFM, as the log of *f*O_2_) buffer resulted at QFM-2 (reducing conditions). Literature studies (cf.^42^ for a review) have considered more oxidizing conditions for the oxygen fugacity (*f*O_2_), with QFM between QFM and QFM + 1. Nevertheless, a deeper crystallization may require more reducing conditions. Also, to obtain a liquid line of descent fitting the previously unrecognized high values of TiO_2_ (> 4 wt.%) of the EF products, we must inhibit the crystallization of Ti-oxides, trying to maintain Ti in the liquid phase. This occurs when the *f*O_2_ is low, as confirmed by the best fit of EF samples with the liquid line of descent with the considered QFM-2 (Table 1; Fig. 5).

**Table 1:** Range of tested parameters and best-fit magma storage conditions for EF and MSC products constrained via Rhyolite-MELTS models.

	Eruptive fissure (EF) magmas	Monogenetic scoria cones (MSC)
**Range of tested parameters**		
Temperature range (°C)	1331–1000	1151–1000
Temperature step (°C)	2	2
Pressure (MPa)	350–900	350–200
Dissolved H2O content (wt.%)	1–2	0.2–1.5
*f *O2 (log units relative to QFM)	QFM-2–QFM+1	QFM–QFM-1
Runs	250	200
**Rhyolite-MELTS best-fit**		
Pressure (MPa)	510	300
H2O content (wt.%)	1.5	1
*f *O2 (log units relative to QFM)	QFM-2	QFM-1
**Petrological constraints**		
	(I) Lack of Eu negative anomaly suggests absence of plagioclase crystallized from the melt	Porphyritic (15–35 vol.%) and plagioclase- dominated. Shallower conditions of crystallization are suggested from petrography.
	(II) Aphyric	
	(III) Most undegassed olivine-hosted melt inclusions present 500 MPa and H2O = 1.5wt.%	

Results suggest that Metehara and Kone-Kokoro EF compositions are explained with an isobaric deep (~ 19 km) crystallization and fractionation of a mafic mineral assemblage (clinopyroxene 11–18%, olivine 10–12%, Ti-magnetite 1–3%), which increases at 43–51 vol.% (clinopyroxene 20–25%, olivine 19–21%, Ti-magnetite 3–5%) for Koka EF products (Table 1). The higher fractionation degree of the southern EF of Koka with regard to the other EF products could be attributed to a higher evolution of magma, possibly related to the southward lithospheric thickening and decrease in extension^19^. Furthermore, samples of Koka EF do not perfectly fit with the obtained liquid line of descent (Fig. 5), a feature possibly resulting from their slightly higher crystal content (up to 5 vol.%) with respect to the Metehara and Kone-Kokoro EF products. For example, the lower P-content of Koka EF with respect to the liquid line of descents could be attributed to the fractionation of small amounts of apatite, conversely to the Metehara EF samples, which are strongly enriched in P_2_O_5_, probably for unfavourable conditions for the fractionation of apatite. In general, Fo_81_ olivine starts to crystallize at 1253 °C, whereas augitic clinopyroxene appears at 1161 °C, decreasing MgO, CaO and FeO (Fig. 5). Therefore, this first modelled configuration of the plumbing system through Rhyolite-MELTS suggests that the scarcity of crystals in EF products can be attributed to an efficient fractionation of mafic crystals from the liquid phase at the base of the crust and direct ascent towards the surface.

The second configuration of plumbing system modelled with Rhyolite-MELTS considers a further step of crystallization before reaching the surface. This should explain the extensive crystallization of plagioclase only in Monogenetic Scoria Cones products, allowed by favourable P–T-H_2_O conditions, and the distinct geochemical signature between MSC and EF. Indeed, geochemical whole rock data and the first step of crystal fractionation highlight that MSC compositions are not primary, but underwent a deep (ca. 510 MPa) fractionation of a mafic mineral assemblage before rising (Table 1). Therefore, after experiencing the previously modelled first step of deep crystal fractionation, magma rose adiabatically to mid-crustal levels and started to crystallize. Due to the plagioclase-dominated assemblage, this mid-crustal reservoir should be located above the depth of nucleation of this mineralogical phase. Results of this second step of simulations derive from ~ 200 runs (conditions in Table 1). The starting composition of this new simulation is the liquid composition after nucleation and growth of the three mafic mineralogical phases in the deep reservoir (olivine 12 vol.%, clinopyroxene 6 vol.% and Ti-magnetite 1 vol.%; ESM 2); the relative starting temperature is 1151 °C (Table 1). Pressures of magma storage between 350 and 200 MPa (13–7 km of depth with density of 2800 kg/m^3^) were tested. The content of dissolved water was set and tested between 1.5 and 0.2 wt.%, simulating a magma releasing most of its volatiles during the deep storage and ascent toward mid-crustal levels. QFM buffer are in the interval QFM—QFM-1, simulating more oxidizing conditions (Table 1). The resulting best-fit liquid line of descent for the rather dispersed composition of MSC products is obtained for 300 MPa (~ 11 km depth assuming an average crustal density of 2800 kg/m^3^), 1.0 wt.% of H_2_O, QFM-1 (dashed lines in Fig. 5B–H; Table 1). Therefore, simulations suggest that compositions of Monogenetic Scoria Cones are explained by the sum of a deep crystal fractionation of clinopyroxene, olivine and Ti-magnetite, plus shallow crystallization of a total mineral cargo of 9–42% (clinopyroxene 4–19%; plagioclase 2–12%; olivine 2–6%; Ti-magnetite 1–5%). The scattered compositions of MSC products with respect to the calculated liquid line of descent could be attributed to a non-efficient crystal fractionation in a deep reservoir, resulting in a crystal cargo between 15 and 35 vol.% and also in small whole-rock compositional differences (Fig. 5; ESM 2). Therefore, this step of crystal fractionation suggests that magmas producing MSC result from an intermediate depth (~ 11 km) storage and crystallization.

## Discussion

**Figure 8:**
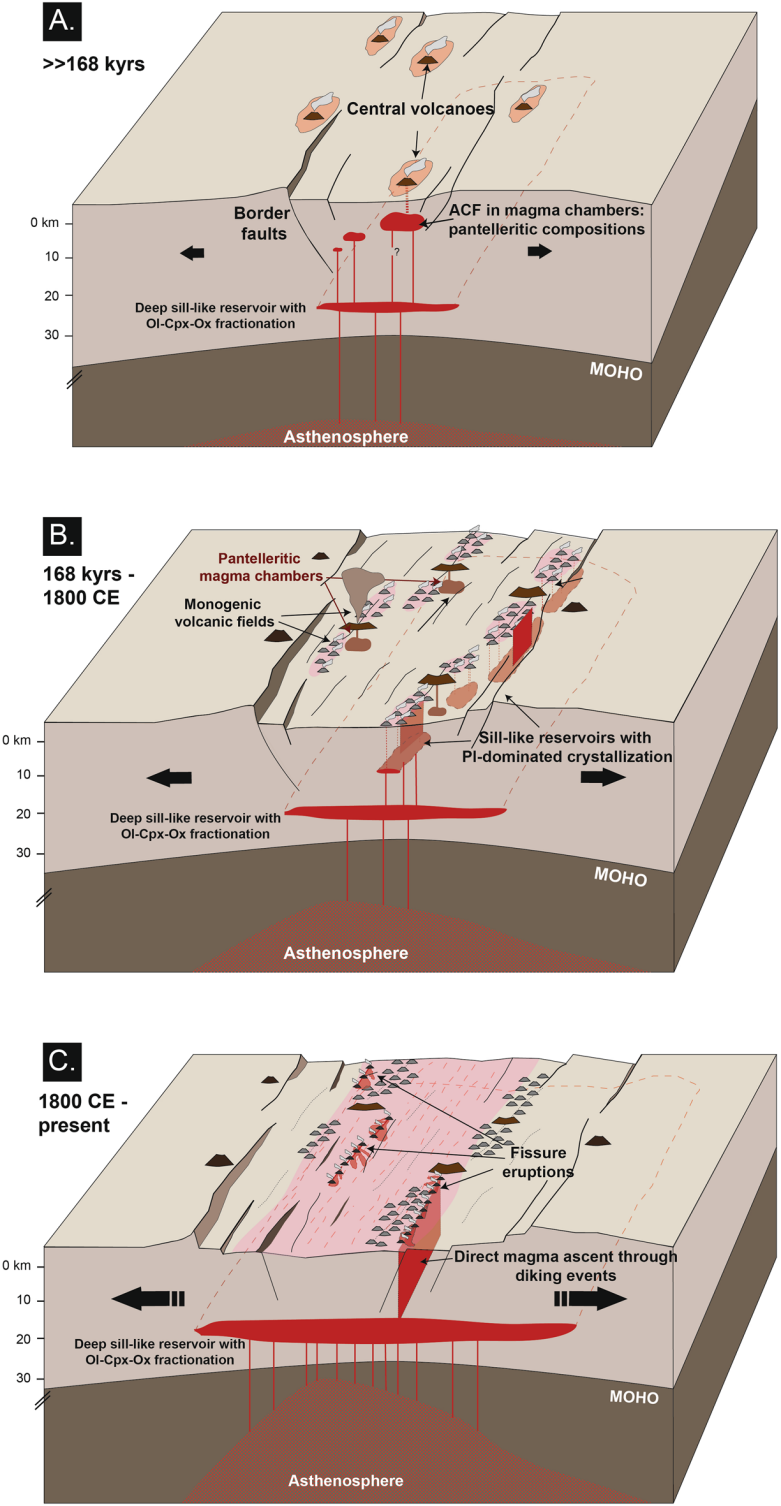
Evolutionary model for the plumbing system of the Northern Main Ethiopian Rift. **(A)** Development of the Northern MER and volcanic activity mainly through central volcanoes. After magmas have been generated down in the Astenosphere, during adiabatic ascent they are stored at different crustal levels before reaching the surface. This promoted magma differentiation towards rhyolitic/pantelleritic compositions (cf.^18,20,41–43^) associated with caldera-forming events. Such plumbing system configuration can be related to immature phases of rifting with lower extension rate and is probably currently represented by the configuration of the plumbing system in the Southern MER. The last caldera-forming event at Fantale is dated at 168 ka^27^. *ACF* assimilation + crystal fractionation. (**B)** Between at least 168 ka and approximately two centuries ago the deep magma recharge below the central volcanoes halted, with the shallow magma reservoirs beneath central volcanoes forming solidifying crystal mushes. Volcanic activity occurred only in the monogenetic scoria cones (MSC). The feeding magma, after having undergone crystal fractionation of the mafic mineralogical phases at the base of the crust, rose up and ponded at mid-crustal levels (ca. 11 km) within sill-like reservoirs. Here, plagioclase-dominated crystallization started, with the rise of small pulses of magmas (either through dykes or drops) producing the monogenetic basaltic scoria cones (MSC) in the NMER. (**C)** The most recent evolution of the NMER (approximately in the last two centuries) has been characterized by linear volcanism associated with eruptive fissures (EF) fed by dikes directly rising from the base of the crust. The shallow plumbing systems below the central volcanoes are completely solidified.

Monogenetic volcanoes along the MER have been generally considered as deriving from a homogenous tectono-magmatic context, being generally grouped as “basalts s.l.” (e.g.^1,19,27,31,35,37^). Our results rather show that these volcanoes can be distinguished into two groups leading to different volcanic structures (Monogenetic Scoria Cones and Eruptive Fissures) deriving from the same primary magma but different ascent history and distinct pre-eruptive dynamics (Fig. 8). Our modelling shows that magmas of Monogenetic Scoria Cones (MSC) derive from the superimposition of deep crystallization and fractionation at lower crustal depths (~ 19 km), in common to eruptive fissure (EF) magmas, and a plagioclase-dominated storage at mid-crustal depths (~ 11 km).

Evidence for a mid-crustal magma reservoir for the MSC mainly results from interpreting plagioclase textures and their core-to-rim compositional profiles. Oscillatory zoning in plagioclase crystals is classically related to crystallization under quiet conditions, i.e. without significant variations of P–T–X-H_2_O. In this context, the ΔAn_1–3_ oscillations in the MSC plagioclases result from crystallization kinetics at the crystal/melt interface, whereas ΔAn_3–7_ oscillations can be attributed to the ponding and movements of plagioclase crystals within a rather chemically and physically homogeneous reservoir^49–51^. Conversely, the resorption textures at the core of the plagioclase could be attributed to high decompression rates in water-undersaturated portions of the feeding system, where considerable amounts of water are still dissolved in the melt^52–54^. The dominance of oscillatory-zoned plagioclases (> 90% of crystals in our dataset) suggests that MSC magmas crystallized in reservoirs with stable chemical and physical conditions or only with minor P–T gradients. The absence of crenulated or resorbed surfaces and of resorbed rims or sieve-textures in the analysed plagioclase of the MSC, together with flat-to-decreasing compositional profiles for An and FeO, indicates that the MSC derive from a “one-shot” intrusion from the crust base, not followed by further magma/gas recharges or rejuvenations. Therefore, MSC magmas rose, with limited crystal fractionation (< 12 vol.%), from the base of the crust and emplaced at ~ 11 km depth, likely feeding sub-horizontal intrusions (as sills; Fig. 8B), adding a further step of (plagioclase-dominated) crystallization. These sill-like intrusions would spatially mimic at depth the high-density MSC volcanic fields indicated in Fig. 1 (black dotted ellipses; Fig. 8B). These hundreds of scoria cones would then derive from the “one-shot” ascent of small magma batches from this mid-crustal reservoir (Fig. 8B). This scenario of stepwise storage is also consistent with previous studies suggesting a ~10 km depth rooting of the feeding structures of monogenetic scoria cones in the MER^55^. Below this depth, the crust is modified by dense gabbroic intrusions (cf.^21^), likely the solidified magma reservoirs feeding the MSC. A simple and likely driving mechanism for this scenario may be lithospheric thinning and related decompression experienced by the rift axis^19,56,57^.

Conversely, magmas producing the recent Eruptive Fissures in the Northern Main Ethiopian Rift do not show evidence of storage at mid/shallow levels of the crust. Their very low crystal cargo (P.I. 0–5 vol.%) suggests the direct ascent of a magma from a reservoir experiencing only recharge from depth and an efficient crystal settling of the denser mineralogical phases (e.g., olivine, pyroxenes, oxides; Table 1; Fig. 8). The lack of plagioclase and of negative anomalies for Eu in the spider-diagrams of Fantale, Kone-Kokoro and Koka EF (Fig. 6A,B) indicates that the main reservoir of EF magma was located below the depth of nucleation of plagioclase, that is here found at ~ 10 km. Geophysical data below the NMER have highlighted a low-velocity zone at 19–25 km, which is the approximate depth of the Moho for Afar-NMER region^58^. Our modelling envisages a plausible deep magma reservoir of the NMER magma plumbing system located at ~ 19 km depth, where magmas are first stored after adiabatic ascent from the source of partial melting (Fig. 8C). Here, magmas producing the EF would experience fractionation of 22–51 vol.% of a mafic solid assemblage (Table 1). These magmas would then rise directly to the surface through dikes influenced by the regional stress field. Rhyolite-MELTS simulations also show that the total amount of fractionated crystals in the EF increases southward, a feature possibly related to the lithospheric thickening and decrease in extension.

As concerns the polygenetic central volcanoes, no evidence of mixing with any magma, as that potentially left in the felsic chambers below them, has been detected in the EF products (Figs. 4, 5, 6, 7). This indicates that the magma rising through dikes from the lower crust is not affected by the composition of these evolved magma reservoirs, even when erupting within a caldera, as at Kone. At the same time, our data confirm that the magma chambers below the polygenetic volcanoes have previously promoted magma differentiation, developing more evolved (i.e., rhyolitic) compositions (Fig. 6; ESM 2; cf.^10,18,20,41–43^). The apparent contradiction between the filtering role of magma chambers and the lack of differentiation of the EF magmas successively passing through them may be explained only assuming the lack of any residual mush in the former chambers, suggesting their current overall solidification. This suggests that the most recent activity along the MER consists of the sporadic but fast rise of deep and undifferentiated magma through dikes, in a context of overall solidification of the shallow plumbing systems below the central volcanoes (Fig. 8C). A recently detected rhyolitic dike intrusion occurred in 2015 a few km north-east of Fantale supports this scenario. In fact, although attributed to the Fantale magma chamber^33^, we suggest that this dike could represent the evolved shallow residual of the nineteenth century Metehara diking episode.

Our results suggest that the current volcanism along the NMER is mainly controlled by far-field stress related to plate motion, rather than any local contribution from near-field stresses induced by the magma chambers below the polygenetic volcanoes. The likely quiescence of the magma chambers of the polygenetic volcanoes is also consistent with the fact that, along the NMER, no central volcano has been observed to deform or experience unrest^7,8^. Indeed, the location of the EF suggests that the portion of magmatic system nearest to the dominant polygenetic volcano currently accommodates most of the extension, representing the present focus for the rise of magma. Only the slightly more evolved composition of the products of the southernmost EF (south of Gedemsa) suggests a lower amount of extension. Conversely, MSC fields focus in the less active peripheral parts of the magmatic systems, probably experiencing minor extension (Fig. 1).

The recent and rapid rise of transcrustal dikes feeding EF suggests a different tectono-magmatic behaviour with regard to that previously observed during the activity of the central polygenetic volcanoes and the monogenetic vents (i.e., MSC) along the NMER. The latter were both related to a stepwise rise and emplacement of magma. Whether magma was transferred through vertically or horizontally propagating dikes, it commonly emplaced at the base of magma chambers (below polygenetic volcanoes) or at mid-crustal depths (below monogenetic volcanoes). Conversely, the recent fissural volcanism (i.e., EF) is related to a rapid and continuous rise of magma from the base of the crust through vertically propagating transcrustal dikes. While the preferred location for the rise of magma has remained focused below the polygenetic volcanoes, the mode of rise of the Eruptive Fissures suggests enhanced conditions for dike propagation within the NMER. As parental magma feeding the monogenetic EF and MSC volcanism is similar, these enhanced conditions may be related to increased tensile stress promoted by plate motion, which enhanced the transition from central polygenetic to fissural volcanism in the NMER in the last centuries. Alternatively, or in conjunction, these enhanced conditions may be related to a general increase in magma supply recently experienced by this rift portion. Determining whether these possible tectonic and/or magmatic variations are the expression of a stable or a transient condition is challenging. On the one side, an evolving rift should increase its amount and rate of extension, as well as magma supply, promoting dike injection. On the other side, the Eruptive Fissures recently observed in the NMER may represent a transient fluctuation of a longer-term behaviour dominated by the stepwise rise of magma, as for example suggested by the decades- to thousands of years-long fluctuations in the amount of geodetic extension or erupted volumes along the MER^15,17,27,59^.

Independent of any stable or transient condition promoting the rise and eruption of the EF magmas, our data suggest an at least medium-term (centuries) significant variation in the delivery of magma to the surface within the NMER. This interpretation is supported by the fact that the NMER has passed from dominant central volcanism (polygenetic volcanoes) and stepwise dike propagation (the MSC vents) to transcrustal dike propagation, developing linear fissure volcanism (the EF vents), modulated by the magma-assisted rifting process occurring at the base of the crust. The activity of transcrustal dikes and the solidification of the magma chambers below the central volcanoes explain the apparent contradiction between the widespread presence of magma at depth and its poor evidence at the central volcanoes.

These features are in line with recent observations in the more mature transitional crust of Afar, where rapid dike emplacement drives plate opening along the rift axis, as in the Asal-Ghoubbet Rift in 1978, at Dallol in 2004 and at the Manda Hararo Rift from 2005 to 2009^2,12–14,60,61^. This behaviour is in stark contrast with the tectono-magmatic features of the less-extending Southern MER, still largely related to the activity of polygenetic central volcanoes, also testified by their more frequent unrest episodes^7,8^ and the much more limited appearance of monogenetic vents and related eruptive fissures. The NMER thus shows that a crucial step in the evolution of continental rifts is the transition from central towards linear transcrustal volcanism, a pre-requisite condition towards plate opening and continental breakup.

## Conclusions

Analysis of the monogenetic volcanism along the NMER reveals two different processes of magma ascent from the lower crust. Most of the monogenetic vents (MSC) reveal a stepwise rise of magma to the surface, characterized by storage at middle crustal levels. A fewer longer eruptive fissures (EF) have formed in the last centuries and are characterized by the direct rise of magma, without evolution or contamination, even when passing through older polygenetic volcanoes, highlighting the rapid rise of transcrustal dikes through frozen plumbing systems. It is not possible to determine whether this variation marks the onset of a major change within the NMER or if it represents only a transient stress fluctuation during the reorganization of a rift. In any case, this variation suggests a transition from central polygenetic to linear fissural monogenetic volcanism possibly promoted by increased tensile conditions and modulated by the magma at the base of the crust, consistently with the behaviour of more mature rifts.

## Methods

### Field analysis and sampling

All data presented here derive from a field analysis performed along the Northern MER in February 2012. The axial portion of the rift has been surveyed from Fantale volcano to the southern termination of Koka Lake (Fig. 1). The survey was accompanied by sampling some representative outcropping volcanic products. On a total of 135 samples, 35 have been taken from as many monogenetic scoria cones located between Fantale and Gedemsa, 50 from the pre- and post-caldera successions of Fantale, Kone and Boseti (not all included here) and 50 from the recent eruptive fissures of Fantale, Kone and Koka Lake (Figs. 1, 2). For reference, samples of the polygenetic volcanism of Fantale, Kone, Boseti and Gedemsa have been also collected and analysed. Among the three volcanoes, most of samples of Kone and Gedemsa resulted too altered to be considered (up to 20 samples with L.O.I. > 4 wt.%), whereas at Fantale an analysis of its northern intra-caldera wall and its caldera floor products is provided (ESM 2). Location of samples are highlighted in Figs. 1 and 2 and their GPS coordinates are given in the Electronic Supplementary Material (ESM 1).

Using satellite images (Google Earth Pro; images acquired in November 2019), 283 monogenetic scoria cones have been identified in the study area, in agreement with previous studies^32^. Among these, 222 lie between Fantale and Boseti volcanoes, whereas 61 lie between Boseti and Gedemsa (cf. Fig. 1 and ESM 1). By using a clustering method similar to that used by^55^, at least three cones aligned along a fracture/fissure and horizontally offset no more than 100 m are considered coeval, or related to the same eruptive episode.

### Geochemical and petrologic analytical techniques

Analyses for obtaining whole rock major element data were performed on powder pellets of all the collected samples at the Dipartimento di Biologia, Ecologia e Scienze della Terra of the University of Calabria (Italy) by means of a Philips PW2404 WD-XRF, taking into account the matrix effect corrections. Gravimetric methods were used for obtaining loss on ignition on each sample, and it was then corrected for Fe^2+^ oxidation. Major elements for whole rocks are reported in ESM 2.

Whole rock trace element compositions were performed at the SGS Laboratories of Toronto (Ontario, Canada) by means of a Perkin Elmer ELAN 6100 inductively coupled plasma mass spectrometer. The first step of analysis is the fusion (within graphite crucible) of powdered rock samples by Na-peroxide, with consequent dissolution using dilute HNO_3_. Calibration of instruments was performed at the beginning and end of each batch of 5 samples by means of 4 runs on international certified reference materials. Precision and accuracy is better than 5% for all analysed elements. The complete major and trace element dataset for the analysed samples is available in ESM 2.

Polished petrographic thin sections have been prepared on a selected and representative number of 50 samples, on which petrographic and mineralogical analyses were conducted. In order to understand the relationships between the MSC and the more recent EF, on 12 selected plagioclase crystals textural and compositional observations were performed, the last also with the execution with step of analysis varying from 5 to 10 μm, depending on the crystal size (ESM 3). Textural observations were instead performed by means of high-contrast back-scattered electron (BSE) images. Both investigations were accomplished at the Dipartimento di Scienze Biologiche, Geologiche e Ambientali of the University of Catania (Italy) with a Tescan Vega-LMU scanning electron microscope coupled with an EDAX Neptune XM4-60 EDS micro-analyser equipped with an ultra-thin Be window and an EDAX WDS LEXS spectrometer (wavelength dispersive low energy X-ray spectrometer) calibrated for light elements. Operating conditions for obtaining high contrast BSE images were set at 20 kV (accelerating voltage) and ~ 8 nA (beam current) and 20 kV and 2 nA for the analysis of major element abundances. Analyses were repeated, during the analytical runs, on internationally certified standards (An-rich plagioclase and glass), giving a precision in the order of 3–5% and an accuracy of 5%.

### Primary magma modelling

In order to obtain the potential primary liquid related to the products of the monogenetic activity, the PRIMELT3 MEGA.XLSM algorithm was used^62^. This provides a mass balance-like solution, based on the olivine addition to the composition of a basic lava until a primary magma composition is reached. As an input to PRIMELT3, the starting compositions have been chosen among the most basic and less porphyritic lavas of each dataset [FK3 (P.I. = 1 vol.%) for MSC and ME14A (P.I. = 12 vol.%) for EF; ESM 2]. Considering these samples as representative for the differentiated liquid phase, resulting compositions are shown in Fig. 5 (purple and yellow stars) and Table 1. In order to have a double check on the obtained primary compositions, a mass balance calculation approach has been also used. Any process of crystal fractionation into the magma reservoir has been reset by adding variable amounts of solid fractions of olivine, clinopyroxene, plagioclase, orthopyroxene and oxides to the same most basic and less porphyritic lavas within each investigated suite, until the resulting crystallizing olivine reaches a composition in equilibrium with the in mantle (with inferred Fo_88-90_). The added mineral compositions are among the most basic of the entire dataset (ESM 4).

### Rhyolite-MELTS modelling

Rhyolite-MELTS is a thermodynamic-based software^46,47^ here used to simulate crystal fractionation processes from a primary magma composition. The best-fit of the resulting liquid lines of descent (LLD) with whole rock compositions of the considered volcanic products (Fig. 5) can provide useful hints on the chemical/physical conditions (P–T–*f*O_2_–H_2_O) of crystallization of the magma before eruption. Although this software may be affected by some limitations, mainly concerning the modelling of some elements (e.g., CaO, P_2_O_5_), felsic compositions and high H_2_O contents^20,47,63^, it still remains a very helpful and popular tool. In particular, the new version of the algorithm used here (v. 1.2.0) results in a good modelling of alkaline basic to intermediate magmas, independently of the geodynamic setting^47^. Therefore, Rhyolite-MELTS is used here only for explaining basic/intermediate compositions and their physical/chemical conditions of crystallization (and not for the peralkaline compositions of the central volcanoes), with the results integrated with the other collected petrographic/mineralogical and geological evidence. The best-fitting liquid lines of descent for the considered suites of samples have been determined (Fig. 5) through more than 450 runs of Rhyolite-MELTS simulations, within the spectrum of parameters indicated in Table 1. The obtained LLDs have been then plotted in the compositional diagrams together with the investigated sample suits, in order to choose the model for which the natural compositions are reproduced. The interval of temperature for the simulation has been set at 2 °C, as larger intervals would have influenced the resolution of fractional crystallization models^42,47^.

## Supplementary Information

Supplementary Information 1.

Supplementary Information 2.

Supplementary Information 3.

Supplementary Information 4.
